# Protein *S*-palmitoylation in immunity

**DOI:** 10.1098/rsob.200411

**Published:** 2021-03-03

**Authors:** Tandrila Das, Jacob S. Yount, Howard C. Hang

**Affiliations:** ^1^ Laboratory of Chemical Biology and Microbial Pathogenesis, The Rockefeller University, New York, NY 10065, USA; ^2^ Department of Microbial Infection and Immunity, The Ohio State University, Columbus, OH 43210, USA; ^3^ Departments of Immunology and Microbiology, Chemistry, Scripps Research, La Jolla, CA 92037, USA

**Keywords:** *S*-palmitoylation, adaptive immunity, innate immune receptors, innate immune effectors

## Abstract

*S*-palmitoylation is a reversible posttranslational lipid modification of proteins. It controls protein activity, stability, trafficking and protein–protein interactions. Recent global profiling of immune cells and targeted analysis have identified many *S*-palmitoylated immunity-associated proteins. Here, we review *S*-palmitoylated immune receptors and effectors, and their dynamic regulation at cellular membranes to generate specific and balanced immune responses. We also highlight how this understanding can drive therapeutic advances to pharmacologically modulate immune responses.

## Graphical abstract

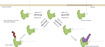

## 1. Introduction

**Figure 1.**
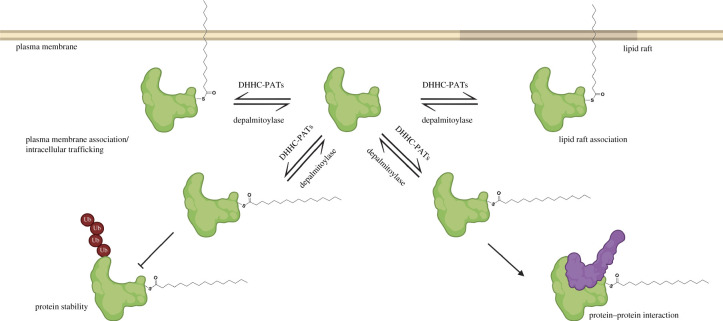
Protein *S*-palmitoylation and regulation. Palmitoylation-depalmitoylation cycle regulates protein-membrane association, lipid raft targeting, protein stability and protein–protein interactions, among others. Dynamic *S*-palmitoylation is mediated by DHHC palmitoyl acyl transferases (DHHC-PATs) and depalmitoylases. Image created with Biorender.com.

*S*-palmitoylation is a posttranslational modification of proteins with lipids. It typically involves the addition of a 16-carbon palmitic acid to cysteines of a protein via a thioester bond (figure 1), but other fatty acids like myristic acid and oleic acid can also be added [1]. *S*-palmitoylation and other lipid modifications control protein-membrane association and trafficking, thereby playing critical roles in protein function and cell signalling [2]. *S*-palmitoylation is unique among lipid modifications because the high energy thioester bond between the fatty acyl group and cysteine residue allows it to be a reversible modification and therefore can impart spatio-temporal control of protein function [3]. For example, this dynamic fatty acid modification of H- and N-ras enables the protein cycling between the plasma membrane and Golgi apparatus, thus maintaining subcellular compartmentalization for diversification of signal transduction (figure 1) [4]. In addition to protein targeting to different compartments and membrane microdomains, the role of *S*-palmitoylation is also implicated in protein stability, conformation, and homotypic and heterotypic interactions (figure 1). *S*-palmitoylation can exert multiple effects simultaneously to orchestrate the desired protein function. Moreover, it can act in concert with other co- and posttranslational modifications to regulate protein functions. Reported *S*-palmitoylated proteins include ion channels, receptors, transporters, enzymes, cell-adhesion proteins, innate immunity effectors and many others. Overall, *S*-palmitoylation is involved in a diverse array of physiological processes, including cellular signalling, differentiation, transcriptional regulation, metabolism and others [5–7].

*S*-palmitoylation of proteins is mediated by palmitoyl acyltransferases (PATs) in intracellular compartments including the endoplasmic reticulum, Golgi apparatus and plasma membrane [8]. In humans, PATs are a family of 23 proteins with a characteristic Asp-His-His-Cys (DHHC) domain essential for catalysis. PATs were first discovered in yeast and are conserved across all eukaryotes, though their numbers and specificities can differ between species [9,10]. Regulation at transcriptional, translational and posttranslational levels, as well as PAT variable domains, determine localization, specific substrate profiles and functionality of individual enzymes [11]. Recent structural studies of PAT family members have provided key understanding of reaction mechanism, substrate recognition, interaction, binding and fatty acyl chain selectivity [12,13]. Genetic, cell-based and animal-based studies have implicated PATs and PAT substrates in many patho-physiological conditions including cancer and schizophrenia [14,15].

Removal of palmitate from proteins is regulated by *S*-depalmitoylases, which catalyse hydrolysis of thioesters [16]. Acyl-protein thioesterase 1 (APT1; also known as lysophospholipase 1, LYPLA1) and acyl-protein thioesterase 2 (APT2; also known as lysophospholipase 2, LYPLA2) were the first identified depalmitoylases for G proteins [17]. Additionally, *α*/*β*-hydrolase domain-containing protein 17 (ABHD17) and other serine hydrolase superfamily proteins were identified as depalmitoylases for PSD-95 and oncogenic protein N-Ras [18,19]. Recently, ABHD10 was identified as a depalmitoylase for peroxiredoxin (PRDX5), a key antioxidant protein, and shown to regulate its antioxidant capacity [20]. These findings provide evidence for roles of depalmitoylases in patho-physiological conditions including cancer and made them important targets for potent inhibitor development [21]. Palmitoyl-protein thioesterase 1 (PPT1), an endo-lysosomal protein, is the first known depalmitoylating enzyme to be linked to a fatal genetic lysosomal storage disorder in neurons, neuronal ceroid lipofuscinoses (NCLs) [22]. Development of inhibitors and chemical probes has advanced our understanding of individual depalmitoylase regulation and substrate specificity [23–25].

**Figure 2.**
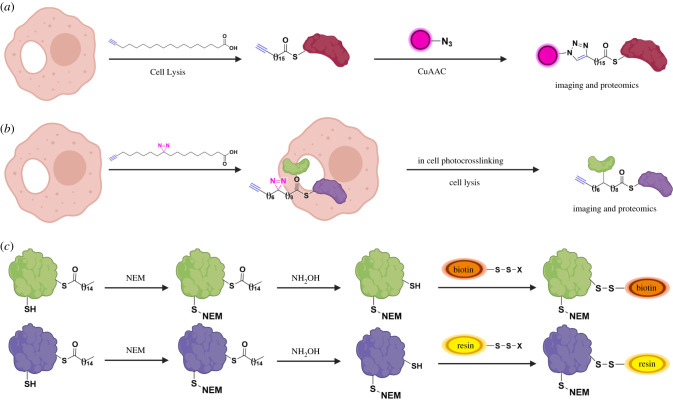
Chemical methods for the study of *S*-palmitoylation. (*a*) Metabolic labelling of cells with palmitic acid reporter alk-16/ODYA and further copper catalysed azide-alkyne cycloaddition (CuAAC) reaction with azido-modified fluorescent or affinity tags for fluorescent visualization or affinity enrichment of *S*-palmitoylated proteins. (*b*) Metabolic labelling with a bifunctional fatty acid chemical reporter and in cell photocrosslinking allows detection and proteomic identification of *S*-palmitoylated protein interactome. (*c*) ABE and acyl-RAC methods involve capping of free cysteines with thiol-reactive reagent like *N*-ethylmaleimide (NEM) followed by removal of *S*-palmitoylation with NH_2_OH. Newly exposed cysteines are then reacted with a thiol-reactive biotin or resin, respectively. Further enrichment allows identification of *S*-palmitoylated proteins. Image created with Biorender.com.

The earliest methods of detection of protein palmitoylation involved metabolic labelling of proteins with radioactive ^3^H, ^14^C or ^125^I containing palmitic acids. These had many limitations including low sensitivity. Development of chemical approaches to study protein *S*-palmitoylation have revolutionized the understanding of the field (figure 2) [26–28]. Palmitic acid chemical reporters, including palmitic acid-like molecules containing azido or alkynyl groups (e.g. alk-16/ODYA), can label cysteines of target proteins using endogenous palmitoylation machinery (figure 2*a*). Using bioorthogonal ligation methods, they can be reacted with fluorophores for visualization or with biotin for enrichment and identification of palmitoylated proteins [29]. Such reporters have further been incorporated in pulse-chase-like experiments to monitor palmitoylation turnover kinetics on proteins [19,30,31]. Moreover, incorporation of photocrosslinking chemical moieties in palmitic acid reporters enabled the identification of an *S*-palmitoylated protein interactome (figure 2*b*) [32]. A complementary chemical approach for the study of *S*-palmitoylation involves modification of thioester linked cysteines in *S*-palmitoylated proteins. This strategy is exploited in methods like acyl-biotin exchange (ABE) and acyl-resin-assisted capture (acyl-Rac) (figure 2*c*) [33,34]. In these methods, free cysteines of palmitoylated proteins are capped and thioester linkages are subsequently cleaved to generate new thiols. These thiols are then selectively labelled by biotin for ABE or thiol-reactive resin for acyl-Rac, allowing further enrichment and detection of palmitoylated peptides or proteins. A variant of this method called acyl-PEG exchange (APE) exploits PEG labelling of newly generated thiols as a mass tag for mobility-shift based assays to identify levels of protein *S*-palmitoylation [35]. Though the above chemical approaches each have individual limitations, they have been successfully applied for global profiling of palmitoylated proteins in yeast, protozoan and mammalian cells [36–39]. Furthermore, identification of *S*-palmitoylomes helped in the development of *in silico* predictive programmes for palmitoylation sites in proteins despite the lack of a consensus motif [40]. Other methods developed for the analysis of specific palmitoylated proteins include in-cell imaging based on bioorthogonal fatty acid labelling and *in situ* proximity ligation or quantification of *S*-palmitoylation levels by gas/liquid chromatography and mass spectrometry [41,42]. Moreover, chemical synthesis or semi-synthesis of modified peptides/proteins has been important for mechanistic understanding of *S*-palmitoylated proteins, as has been exemplified by the classical study on palmitoylated Ras isoforms [4,27,28]. Recent reports of site-specific chemical modifications of Ras and transmembrane protein IFITM3 with palmitate mimics in live cells and evaluation of activity afford new opportunities to study *S*-palmitoylation and other lipid modifications in live cells [43,44].

We previously reviewed the application of these chemical tools for global profiling of palmitoylated proteins in immune cells including T cells, dendritic cells, macrophages and B cells [45]. Since the publication of this review, there has been remarkable progress in identifying new palmitoylated immunity proteins, and also a renewed emphasis on functional characterization of individual *S*-palmitoylated immune proteins. In this review, we will highlight mechanistic studies on the roles of *S*-palmitoylation in the regulation of immune protein function in immune cells (table 1). This review is organized by proteins involved in adaptive and innate immune pathways (i.e. immune sensors, signal transducers, signal regulators and immune effectors). There are excellent reviews that describe the role of protein *S*-palmitoylation in other physiological processes [79].

**Table 1.** *S*-palmitoylated immunity-associated proteins.

	DHHC-PATs/ APTs	site of modification	method of study	function of modification	ref.
**T cells**					
CD4		C394, C397	radiolabelling	lipid raft association and clustering	[46,47]
CD8			radiolabelling	CD8 association with kinase Lck	[48]
Lck	DHHC2, 21	C3, 5	radiolabelling	lipid raft association	[49,50]
Fyn	DHHC2, 3, 7, 10, 15, 20, 21	C3, 6	radiolabelling	plasma membrane localization	[51]
LAT		C26, C29	radiolabelling	lipid raft association protein stability	[52,53]
Fas	DHHC7	C199	ABE	lipid raft association	[54]
FasL	DHHC7	C82	ABE	lipid raft association	[55]
PD-1	DHHC9	C192	alk-16	protein stability	[56]
**B cells**					
CD81		C6, C9, C80, C89, C227, C228	radiolabelling	protein interaction	[57]
HGAL		C43, C45	radiolabelling	lipid raft association	[58]
**immune receptors and adapters**					
PD-L1	DHHC3			protein stability	[59]
TLR2	DHHC2, 3, 6, 7, 15	C609	alk-16	plasma membrane localization	[60]
MyD88	DHHC6	C113, C274	ABE	binding of IRAK4 to MyD88	[61]
STING	DHHC3, 7, 15	C88, C91	radiolabelling	membrane clustering	[62]
NOD1/2	DHHC5	NOD1 C558, C567, C952 NOD2 C395, C1033	ODYA, ABE	membrane association	[63]
FCGR2A		C208	radiolabelling	lipid raft association	[64]
ASAP2	DHHC6	C86	ABE	regulation of phagocytosis	[65]
CD36	DHHC 5 APT1	C3, C7, C464, C466	Acyl-RAC	fatty acid uptake	[66,67]
β1-adrenergic receptor		C392, C393, C414	radiolabelling/alk-16		[68]
CCR5		C321, C323, C324	radiolabelling	protein trafficking	[69]
S1PR1	DHHC5			protein–protein interaction	[70]
IFNAR1		C463	radiolabelling	downstream STAT phosphorylation	[71]
JAK1		C541, C542	RAC like assay	plasma membrane association	[72]
STAT3	DHHC7 APT2	C108	alk-14/ ABE	differentiation of T_H_17	[73]
TNFR1	APT2	C248	Acyl-RAC, ODYA	protein stability	[74]
**immune effectors**					
IFITM3	DHHC3, 7, 15, 20	C71, C72, C105	alk-16	antiviral activity	[35,75]
TNFα		C47	radiolabelling	plasma membrane association	[76]
DR6		C368	radiolabelling		[77]
DR4		C261, C262, C263	radiolabelling	lipid raft association and oligomerization	[78]

## 2. *S*-palmitoylation of proteins in adaptive immunity

**Figure 3.**
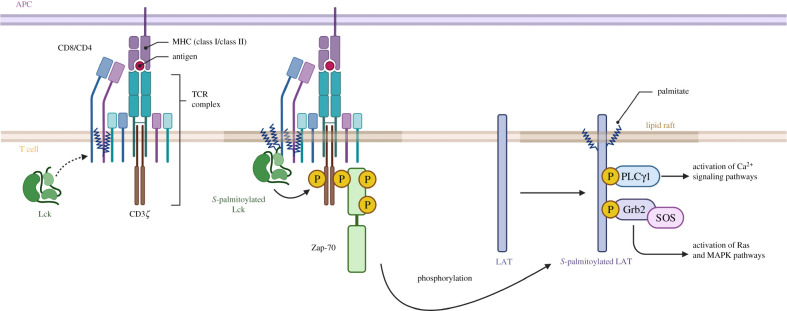
*S***-**palmitoylated proteins in T cell signalling. *S*-palmitoylated Src kinases (Lck) phosphorylates TCR complex leading to ZAP-70 recruitment and activation. Activated ZAP-70 phosphorylates palmitoylated LAT leading to downstream signalling pathways. Image adapted from ‘TCR Downstream Signalling’ by BioRender.com.

*S*-palmitoylation plays an important role in the regulation of host adaptive immune responses. Multiple studies investigated the *S*-palmitoylome of T cells using different methods and identified important factors involved in T cell activation and signal transduction (figure 3) [31,37,80–82]. The first step in T cell receptor (TCR) signal transduction involves the binding of TCR to peptide–major histocompatibility complexes (MHCs) on antigen-presenting cells (APCs). This binding step is followed by recruitment of Src family protein tyrosine kinase Lck to the cytoplasmic domains of CD4 and CD8 co-receptors. This triggers a signalling cascade leading to the formation of the LAT signalosome. Further signal propagation happens via Ca^2+^–calcineurin, mitogen-activated protein kinase (MAPK) and nuclear factor-κB (NF-κB) signalling pathways.

### 2.1. TCR coreceptors

*S*-palmitoylation of TCR subunits has not been reported, but radio labelling studies identified TCR coreceptor CD4 palmitoylation. CD4 is palmitoylated at Cys394 and Cys397, at the junction of the transmembrane and cytoplasmic domain [46]. CD4 *S*-palmitoylation regulates clustering with TCR/protein kinase C (PKC) *θ* in lipid rafts [47]. Coreceptor CD8 is a heterodimer of CD8*α* and CD8*β*. In humans, both CD8*α* and CD8*β* are *S*-palmitoylated, whereas in mice only CD8*β* is *S*-palmitoylated. Mouse CD8*β S*-palmitoylation is necessary for efficient CD8 coreceptor function as it increases CD8 association with Lck in lipid rafts [48].

### 2.2. Src family kinases

Src family kinase Lck is *S*-palmitoylated at Cys3 and Cys5 and this promotes its plasma membrane association [49,83]. Interestingly, Lck N-myristoylation at Gly2 is required for subsequent palmitoylation. Deletion of Lck *S*-palmitoylation reduces Lck-CD4 association and downstream signalling [84]. Further studies show that site-specific *S*-palmitoylation at Cys3 is important for Lck lipid raft localization [50].

*S*-palmitoylation of Fyn, another Src family kinase involved in T cell signal transduction, has also been shown to play an important role in its membrane association [51]. Fyn can be *S*-palmitoylated at Cys3 and Cys6 but Cys3 is the major site and is the most important for lipid raft association. Activation of Fyn by Lck in lipid rafts leads to TCR/CD3 activation and downstream signalling. DHHC2, 3, 7, 10, 15, 20, 21 have all been shown to mediate Fyn *S*-palmitoylation.

### 2.3. TRAPs

Activation of transmembrane adaptor proteins (TRAPs) is a crucial step in the T cell signal transduction pathway. TRAPs like LAT, phosphoprotein associated with GEMs (PAG) and Lck-interacting membrane protein (LIME) are *S*-palmitoylated and show lipid raft association [85]. LAT activation leads to the recruitment of key molecules for downstream signal propagation. LAT is dually *S*-palmitoylated at a membrane juxtaposed Cys-X-X-Cys motif. *S*-palmitoylation of LAT is important for its stability and plasma membrane association [52,53]. Deletion of LAT *S*-palmitoylation hinders TCR interaction and recruitment of PLC*γ* and Grb2 for downstream signal propagation as evident from impaired calcium influx and Erk activation [86].

Beyond T cell activation, LAT *S*-palmitoylation is also shown to regulate T cell anergy, an inability of previously responsive T cell to respond to TCR stimulation. Anergic T cells show reduced LAT palmitoylation, lipid raft association and immunological synapse recruitment [87]. This should be further explored for therapeutic interventions to inhibit or induce T cell anergy in cancer, transplantation and infectious disease.

### 2.4. Fas and FasL

Apoptosis of T cells that recognize self-antigens is necessary to prevent autoimmune diseases. Fas and Fas ligand (FasL) are critical regulators of T cell apoptosis. Fas binding to FasL leads to recruitment of death-inducing signalling complex (DISC) in Fas-bearing cells which activates caspase-3 mediated apoptosis. Fas is *S*-palmitoylated at single Cys199 in human and is required for its stability [54]. DHHC7 is identified to palmitoylate Fas. Mutation of the Fas *S*-palmitoylation site reduces its lipid raft association and impairs apoptosis signalling.

An ABE-based assay identified FasL *S*-palmitoylation [55]. FasL is palmitoylated at Cys82 located at the N-terminal end of the transmembrane domain. S-palmitoylation modulates FasL lipid raft partitioning and proteolytic cleavage by ADAM10 for efficient induction of Fas-mediated cell death.

### 2.5. PD-1 and PD-L1

Programmed death protein 1 (PD-1) is a T cell surface receptor that, upon activation, suppresses T cell proliferation and cytokine production. PD-1 ligands PD-L1 and PD-L2 are expressed on antigen-presenting cells and tumour cells. *In silico* motif-based prediction identified PD-1 *S*-palmitoylation at Cys192 between its transmembrane and cytosolic domain, which was confirmed by metabolic labelling studies [56]. PD-1 *S*-palmitoylation is catalysed by DHHC9. PD-1 palmitoylation is necessary for its stability. Recent studies show that some cancer cells also express PD-1, thus enabling them to promote tumour growth independent of adaptive immunity. *S*-palmitoylation of PD-1 in tumour cells can modulate downstream mammalian target of rapamycin (mTOR) signalling and proliferation.

PD-L1 expressed on tumour cells is also *S*-palmitoylated and this modification inhibits PD-L1 ubiquitination and trafficking to lysosomes for degradation [59]. DHHC3 catalyse PD-L1 palmitoylation. Inhibition of PD-L1 palmitoylation using 2-bromopalmitate or via DHHC3 silencing increases anti-tumour activity in cells and in mice. These discoveries of PD-1 and PD-L1 palmitoylation and targeting for modulation of T cell immune responses in cancer provide exciting opportunities for combinatorial approaches along with immune checkpoint therapy.

### 2.6. B cells

*S*-palmitoylation of critical proteins in B cells has also been identified. Indeed, an ABE-based profiling of B cells identified many candidate *S*-palmitoylated proteins [80]. However, there have been limited studies on functional implications of B cell protein *S*-palmitoylation.

B cell receptor (BCR) coreceptor CD81 is *S*-palmitoylated at multiple membrane juxtaposed Cys residues [57]. *S*-palmitoylation of CD81 is required for enhanced BCR-coreceptor complex lipid raft association [88]. It is also important for CD81 association with other BCR coreceptors CD19 and CD21 [57]. Inhibition of CD81 *S*-palmitoylation affects recruitment of downstream signalling molecules and activation of kinases PI3 K and PKC.

Human germinal centre-associated lymphoma (HGAL) is an adaptor protein involved in BCR signalling. Radio labelling studies identified HGAL *S*-palmitoylation [58]. *S*-palmitoylation of HGAL regulates binding and activation of Syk kinase for downstream signalling. HGAL *S*-palmitoylation deletion mutant localizes in the cytoplasm and significantly impairs chemoattractant-induced cell motility.

## 3. Palmitoylation of proteins in innate immunity

### 3.1. Innate immune receptors and signalling adapter proteins

**Figure 4.**
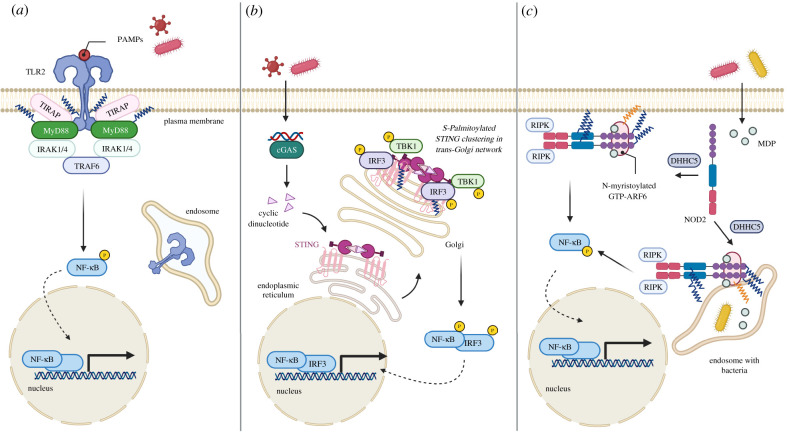
*S*-palmitoylated innate immune receptors and signalling adapter proteins. (*a*) Activation of *S*-palmitoylated TLR2 by pathogen associated molecular patterns (PAMPs) leads to MyD88 signalling. *S*-palmitoylated MyD88 forms a complex with IL-1 receptor-associated kinase (IRAKs) for downstream signalling and subsequent translocation of nuclear factor-κB (NF-κB) to the nucleus for cytokine induction. (*b*) STING binds to cyclic dinucleotides at endoplasmic reticulum and translocates to Golgi apparatus where it is palmitoylated. At the trans-Golgi network, *S*-palmitoylated STING is clustered and recruit TBK1 and IRF3 for downstream signalling. (c) DHHC5 mediated *S*-palmitoylation of NOD1/2 leads to its recruitment to bacteria-containing phagosome. Further exposure to bacteria derived molecules induces NF-κB signalling. Image created with Biorender.com.

The innate immune system responds to microbial invasion by signalling through pattern recognition receptors (PRRs) that bind conserved microbial features or molecules associated with cellular damage. PRRs can be broadly classified into several families of related molecules. These families include the Toll-like receptors (TLRs), C-type lectin receptors, RIG-I-like receptors and NOD-like receptors. Additionally, a receptor known as cyclic GMP-AMP (cGAMP) synthase (cGAS) is involved in microbial DNA detection. Several of these molecules and/or their signalling adapter proteins have been demonstrated to be *S*-palmitoylated in recent years (figure 4). As we will describe below, *S*-palmitoylation, where it has been detected, is generally an activating modification for innate immune signalling through several of these microbial sensing pathways.

#### 3.1.1. TLRs

Toll-like receptors (TLRs) were among the first of the microbe-sensing PRRs to be discovered. TLRs are transmembrane proteins that use leucine-rich repeat domains to detect diverse microbial products, such as proteins, nucleic acids and glycans. The human genome encodes 10 known TLRs, which localize either to the cell surface or within endosomes. Binding of a TLR to its ligand results in signalling through TLR-associated cytoplasmic adapter proteins, including MyD88. TLR signalling generally culminates in activation of the transcription factor NF-κB and production of inflammatory cytokines. *S*-palmitoylome profiling of murine dendritic cells using an alkynyl palmitate analogue with click chemistry-based biotin pulldowns identified TLR2 as a putative palmitoylated protein [60]. *S*-palmitoylation was confirmed and mapped to Cys609 via mutagenesis of candidate cysteines. C609 is located directly adjacent to the cytoplasm-facing side of the TLR2 transmembrane domain. Cys609 mutant constructs showed less responsiveness than WT TLR2 to known TLR2 microbial ligands in NF-κB reporter assays. Further, 2-BP inhibition of TLR2 palmitoylation in murine dendritic cells partially reduced TLR2 levels at the cell surface, and significantly reduced cytokine production in response to TLR2 ligands. Together, these data indicate that TLR2 palmitoylation at Cys609 is required for its proper localization and full antimicrobial activity (figure 4*a*) [60].

*S*-palmitoylation levels on TLR2 were increased by overexpression of several DHHC proteins suggesting possible enzymatic redundancy for this activating modification [60]. Cys609 is highly conserved among TLR2 proteins throughout evolution, further supporting a significant role for this modification in antimicrobial functionality. Several additional human TLRs possess cysteine residues near the cytoplasmic border of their transmembrane domains, including TLRs 1, 5, 6, 7, 8, 9 and 10. However, of the human TLRs, only TLRs 2, 5 and 10 showed robust labelling with alkynyl palmitate analogue, with TLR10 providing the strongest palmitoylation signal relative to total protein levels [60]. Whether *S*-palmitoylation regulates the activity of TLRs 5 and 10, or whether other TLRs are palmitoylated under specific cellular conditions, remain to be determined.

#### 3.1.2. Myd88

MyD88 is a critical signalling adaptor protein for all TLRs, except for TLR3. The discovery of MyD88 *S*-palmitoylation came as a follow-up to the observation that inhibition of fatty acid synthase by the chemical inhibitor C75 reduced inflammatory responses upon TLR2, 4 or 7/8 stimulation of cells [61]. This inhibition of TLR signalling by C75 was pinpointed to MyD88 through studies examining NF-κB activation upon overexpression of various signalling molecules downstream of TLRs. Given that the product of fatty acid synthase is palmitate, the *S*-palmitoylation of MyD88 was investigated and confirmed using chemical reporter labelling and ABE methods [61].

Candidate *S*-palmitoylated cysteines, Cys113 and Cys274, were identified on MyD88 by mass spectrometry, and were further confirmed to be sites of modification via cysteine to alanine mutagenesis of MyD88 constructs and ABE analysis [61]. Interestingly, while single mutations of Cys113 or Cys274 both significantly decreased total MyD88 *S*-palmitoylation, a double mutant did not show a further decrease in palmitoylation. In terms of NF-κB and MAPK activation, mutation of Cys113 decreased responsiveness to the TLR4 ligand LPS, while mutation of Cys274 had no measurable effect. A Cys113 to Ala MyD88 mutant failed to recruit the IRAK4 signalling molecule upon TLR stimulation, indicating that *S*-palmitoylation is required for recruitment of IRAK4 to the Myddosome signalling complex downstream of TLR ligand binding (figure 4*a*). It will be interesting to determine whether MyD88 *S*-palmitoylation promotes localization with TLRs, such as TLR2, that are also palmitoylated [60]. It is also of note that MyD88 *S*-palmitoylation was not detected in any of the published *S*-palmitoyl proteome studies [40], including those done in macrophages and dendritic cells, which may suggest that robust MyD88 *S*-palmitoylation must be induced or that palmitoylated MyD88 exists in poorly soluble protein aggregates.

DHHC6 was identified as a leading candidate palmitoyltransferase for MyD88 based on its ability to increase MyD88 *S*-palmitoylation when overexpressed and based on its high expression in myeloid cell types known to have potent TLR activities [61]. As such, knockdown of DHHC6 decreased MyD88 *S*-palmitoylation and LPS responsiveness of macrophages. Overall, *S*-palmitoylation of MyD88 appears to be a required regulatory modification that can be targeted to decrease TLR-mediated inflammation either by directly inhibiting enzymatic palmitoylation of MyD88 or through limiting endogenous palmitate available for protein modification [61].

#### 3.1.3. STING

The stimulator of interferon genes (STING) is a signalling protein involved in the response to DNA in the cytosol. STING is a multipass transmembrane protein that is activated by the cyclic dinucleotide cGAMP, which is produced by cGAS upon sensing DNA in the cytosol. Ligand-activated STING moves from the ER to the Golgi apparatus and recruits signalling molecules to activate NF-κB and interferon regulator factor 3 (IRF3), resulting in the production of proinflammatory cytokines and type I interferons (IFNs). Mukai *et al*. [62], hypothesized that STING is post-translationally modified at the Golgi and discovered that STING could be modified with radiolabelled palmitic acid upon cellular activation with the chemical STING agonist DMXAA. Further, overexpression of Golgi-localized DHHCs 3, 7 and 15 increased STING *S*-palmitoylation. Conversely, 2-BP treatment of cells eliminated STING palmitoylation and prevented cytokine production upon DMXAA stimulation, suggesting that palmitoylation is necessary for inflammatory cytokine induction by STING. The primary sites of modification on STING were mapped to Cys88 and Cys91. A STING mutant construct with serine substitutions at these positions trafficked from the ER to the Golgi similarly to WT STING upon DMXAA stimulation, but could not induce activation of NF-κB and IRF3, and thus failed to induce proinflammatory downstream gene products, including type I interferons. These results demonstrate that *S*-palmitoylation of STING at specific cysteines is required for its inflammatory signalling function (figure 4*b*) [62].

Activating STING mutations in the human population have been associated with an inflammatory autoimmune interferonopathy known as STING associated vasculopathy with onset in infancy (SAVI). Known STING mutants associated with SAVI lost their ability to spontaneously activate the IRF3- and NF-κB-dependent gene promoters when combined with *S*-palmitoylation-impairing mutations at Cys88 and 91 or when palmitoylation was inhibited by 2-BP treatment of cells [62]. These results suggested that palmitoylation of STING could potentially be targeted therapeutically in SAVI. Indeed, in an attempt to identify STING inhibitors by chemical screening, small molecule inhibitors that covalently react with Cys91 and block palmitoylation were discovered [89]. These inhibitors prevented clustering of STING at the Golgi and prevented STING-dependent inflammation in mouse models. Concurrently, a second group identified endogenously formed nitro-fatty acids as inhibitors of STING palmitoylation via covalent reaction with Cys88 [6]. Nitro-fatty acids were produced during DNA virus infection, suggesting that this may be a natural cellular feedback mechanism to prevent hyperactivation of the cGAS-STING pathway. Further, the addition of nitro-fatty acids to fibroblasts derived from SAVI patients prevented the constitutive type I IFN production by these cells [90]. These exciting developments in small molecule inhibitors of STING provide compelling proof-of-concept studies toward therapeutic targeting of STING palmitoylation [91].

#### 3.1.4. NOD1/2

Nucleotide oligomerization domain (NOD) 1 and 2 proteins are cytosolic innate immune receptors that detect bacterial peptidoglycans. NOD1 and NOD2 are primarily soluble in the cytosol with a portion of these proteins associated with the cellular plasma membrane at steady state. However, they are rapidly redistributed to phagosomal membranes upon intracellular bacterial infection, where they activate NF-κB and MAPK signalling pathways. Since NOD1/2 lack transmembrane regions or traditional lipid-binding motifs, it was hypothesized that their rapid membrane association could be mediated by dynamic *S*-palmitoylation [63]. Indeed, treatment of cells with palmitoylation inhibitor 2-BP altered the localization of both NOD1 and NOD2 as visualized by fluorescence microscopy, a phenomenon that was confirmed by biochemical fractionation techniques to be due to loss of membrane association. 2-BP treatment of macrophages abrogated the ability of the cells to respond to NOD1/2 ligands in terms of NF-κB and MAPK pathway activation. *S*-palmitoylation of NOD1 and NOD2 were both subsequently confirmed by ABE and chemical reporter labelling methods. *S*-palmitoylation of NOD1 was mapped to three cysteine residues, Cys558, 567 and 952. A triple cysteine mutant of NOD1 lost its membrane association and ability to activate NF-κB, an effect that could be rescued by fusion of a known *S*-palmitoylation amino acid motif to NOD1. Similar effects were seen for NOD2, though in this case *S*-palmitoylation was mapped to Cys395 and 1033 (figure 4*c*). A BioID screen for NOD1 and NOD2 interacting proteins identified DHHC5 as a probable candidate for modification of these immune effectors [63]. Knockdown or knockout of DHHC5 resulted in decreased *S*-palmitoylation of NOD1 and NOD2, decreased NOD membrane association, and decreased activation of NF-κB and MAPK pathways in response to NOD ligands. Further, both DHHC5 and intact NOD *S*-palmitoylation sites were required for recruitment of NOD1/2 to *Salmonella*-containing phagosomes [63].

NOD2 variants with decreased peptidoglycan responsiveness have been associated with pathologies, such as Crohn's disease. Five of six disease variants that were tested showed major decreases in *S*-palmitoylation, suggesting that defective palmitoylation-dependent membrane association underlie their decreased functionality [63]. Conversely, a gain-of-function NOD2 variant showed significantly increased baseline *S*-palmitoylation, and 2-BP treatment eliminated its hyperactivation of NF-κB. Overall, it has emerged that *S*-palmitoylation is required for NOD1/2 localization with bacteria-containing membranes and activation of downstream pathways. However, it remains to be determined precisely how bacterial sensing triggers a rapid increase in NOD1/2 *S*-palmitoylation and how this facilitates inflammatory signalling. Recent cross-linking studies with peptidoglycan fragment muramyl-dipeptide (MDP) photoaffinity reporter showed MDP-induced NOD2 interaction with the plasma membrane or endosome resident N-myristoylated GTP-ARF6 [92]. It will therefore be interesting to understand the role of NOD2 palmitoylation in mediating interactions with membrane-bound host proteins.

#### 3.1.5. Phagocytosis receptor

Phagocytosis for engulfing of pathogens or foreign particles by immune cells also involves initial surface receptor-mediated recognition. Phagocytosis receptor FCGR2A is *S*-palmitoylated and regulates its lipid raft association [64]. Furthermore, *S*-palmitoylation of FCGR2A associated kinase ASAP2 can regulate FCGR2A mediated phagocytosis [65].

S-palmitoylation of scavenger receptor CD36 has also been well studied. CD36 is a transmembrane glycoprotein receptor expressed on immune cells including monocytes, macrophages and other cell types, such as adipocytes and cardiac myocytes. CD36 binds to and mediates uptake of oxidized phospholipids, oxidized lipoproteins and long-chain fatty acids, thus playing a major role in lipid homeostasis in cells. In addition, CD36 plays an important role as an innate immune sensor that can recognize and bind to bacterial cell wall components, erythrocytes infected with *Plasmodium falciparum* and apoptotic cells. *S*-palmitoylation of CD36 by DHHC4 and DHHC5 was shown to play a regulatory role in adipose tissue fatty acid uptake in mice [66]. Binding of *S*-palmitoylated CD36 to fatty acids activates the kinase Lyn. Activated Lyn phosphorylates DHHC5 and inhibits its *S*-palmitoylation activity. Depalmitoylation of fatty acid bound CD36 by APT1 leads to downstream signalling to initiate endocytosis of fatty acids. Pharmacological or genetic perturbations of this dynamic palmitoylation–depalmitoylation cycle disrupts fatty acid uptake of cells [67]. Further investigations of CD36 *S*-palmitoylation in downstream immune signalling will be helpful to understand connections between lipid metabolism and inflammation.

Moreover, many members of the G protein-coupled receptor (GPCR) family including *β*_1_-adrenergic receptor, S1P receptor subtype 1 (S1PR1), CCR5 are known to be *S*-palmitoylated [68–70]. CCR5 and other *S*-palmitoylated cell surface receptors like EGFR are also used by viruses like human immunodeficiency virus (HIV) and influenza A virus (IAV), respectively, to enter the host cell successfully. Moreover, It should be noted that *S*-palmitoylation not only plays a role in the host immune system but the host *S*-palmitoylation machinery is also exploited by many viral or bacterial proteins for host infection [93,94]. For example, *S*-palmitoylation of the currently pandemic SARS-CoV-2 spike protein by DHHC5 is important for virus entry in ACE2 expressing cells [95].

### 3.2. Palmitoylation of innate immune effectors

Activation of innate immune receptors initiates signalling pathways resulting in the production of proinflammatory cytokines including tumour necrosis factors (TNFs), IFNs, chemokines and immune effector proteins for the elimination of microbial pathogens.

#### 3.2.1. IFN signalling

IFNs bind to cell surface IFN receptor leading to activation of Janus tyrosine kinase (Jak)/signal-transducing activators of transcription (STAT) pathways to induce expression of IFN stimulated genes (ISGs). Type I IFNs (IFN*α*/*β*) engage the IFNAR receptor complex which is a heterodimer of IFNAR1 and IFNAR2. Radio labelling with [^3^H] palmitic acid identified *S*-palmitoylation of IFNAR1 and IFNAR2 [71]. IFNAR1 is *S*-palmitoylated at Cys463 present in the cytoplasmic domain proximal to the transmembrane domain. Mutagenesis of Cys463 to Ala does not affect receptor stability, endocytosis or intracellular distribution. However, loss of IFNAR1 *S*-palmitoylation results in decreased STAT phosphorylation and nuclear translocation, thereby affecting IFNα-stimulated gene transcription.

**Figure 5.**
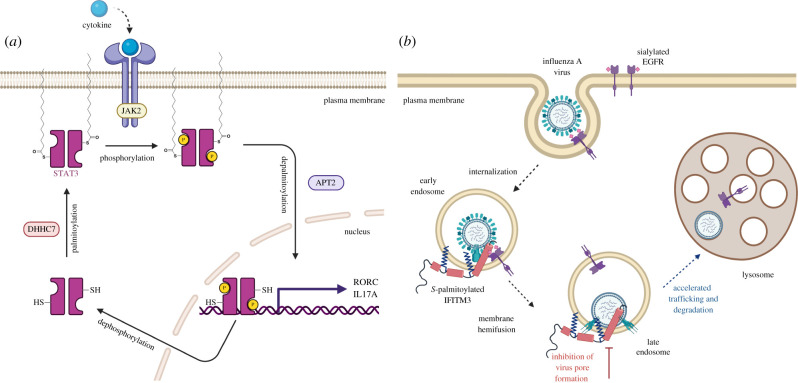
*S*-palmitoylated innate immune effectors. (*a*) STAT3 dynamic *S*-palmitoylation in cells. STAT3 palmitoylation by DHHC7 leads to membrane association and phosphorylation. Depalmitoylation of p-STAT3 by APT2 leads to nuclear translocation of p-STAT3 and expression of STAT3 target genes. (*b*) *S*-palmitoylated IFITM3 restricts enveloped virus entry in cells through the endocytic pathway. IFITM3 colocalizes with virus-containing endosomes and prevents the release of the virus genetic material into the cytoplasm by physically restricting virus membrane pore formation and shuttling the virus for lysosomal degradation. Image created with Biorender.com.

Palmitoylome profiling of adipocytes identified *S*-palmitoylation of four proteins in the JAK/STAT pathway JAK1, STAT1, STAT3 and STAT5A [72]. Palmitoylation of JAK1 and JAK2 was confirmed in adipocytes using an acyl-RAC-like assay. JAK1 palmitoylation was mapped to highly conserved Cys541 and Cys542. Mutation of these two Cys to Ser led to loss of *S*-palmitoylation and impaired JAK1 plasma membrane association. *S*-palmitoylation of STAT family members STAT1*α*, STAT1*β*, STAT3, STAT5B and STAT6 has been demonstrated using metabolic labelling and acyl exchange methods [73]. Canonical type I and type III IFN pathway involves both STAT1 and STAT2 activation and heterodimerization, whereas type II IFN pathway involves STAT2 activation and homodimerization. STAT2 and STAT4 *S*-palmitoylation has not been reported. Recently, STAT3 palmitoylation has been implicated in the regulation of T_H_17 cell differentiation (figure 5*a*) [73]. T_H_17 cells are proinflammatory T cells which express interlukin-17 (IL-17) and retinoic acid receptor-related orphan receptor gamma t (ROR*γ*t). STAT3 undergoes a palmitoylation–depalmitoylation cycle on Cys108 mediated by DHHC7 and APT2, respectively. This cycle accelerates T_H_17 cell differentiation by promoting membrane association, phosphorylation and nuclear translocation of STAT3. Interestingly, phosphorylated STAT3 is selectively depalmitoylated by APT2. Accelerated differentiation of T_H_17 has been connected to autoimmune diseases. DHHC7 and APT2 have been shown to be upregulated in patients with inflammatory bowel disease (IBD). Inhibition of APT2 activity or knockout of DHHC7 in a mouse model relieves IBD symptoms. Inhibition of STAT3 *S*-palmitoylation–depalmitoylation cycle could thus be a potential therapeutic strategy for IBD [73].

#### 3.2.2. IFN effectors

Palmitoylome analysis also identified several ISGs in a murine dendritic cell line and murine embryonic fibroblasts, including bone marrow stromal antigen 2 (BST2) or tetherin, immunity-related GTPase M1 (IRGM1) and interferon-induced transmembrane protein 3 (IFITM3) [60]. We have studied *S*-palmitoylation of IFITM3 and its regulation in our laboratories over the past 10 years. Interferon-induced transmembrane proteins (IFITM1, IFITM2 and IFITM3), which share highly conserved palmitoylation sites, are involved in host immune response to viral infections. IFITM3 is the most active isoform and restricts many viruses including influenza A virus (IAV), dengue virus (DENV), Ebola virus (EBOV), human immunodeficiency virus (HIV), hepatitis C virus (HCV) and Zika virus (ZIKV) [96,97]. IFITM3 also inhibits SARS coronavirus (SARS-CoV) infections [98]. Surprisingly, IFITM3 promotes endemic human coronavirus OC43 infection [99,100]. Recent pseudovirus infection and cell–cell fusion studies provide conflicting results regarding a role of IFITM3 in pandemic SARS-CoV-2 infection [101–104]. Infection studies with genuine SARS-CoV-2 shows IFITM3 acts primarily as an antiviral factor but an endocytosis mutant which localizes to the plasma membrane acts as a proviral factor [105].

IFITMs are present in basal levels in many different cell types. They are intrinsically expressed in embryonic stem cells for protection against virus infections [106]. IFITMs reduce the susceptibility of placental trophoblasts to viral infection, but they also inhibit trophoblast cell fusion, an essential process for placental development mediated by syncytin, which is derived from retroviral envelope [107,108]. Furthermore, the resistance of CD8+ resident memory T cells expressing IFITM3 to IAV infection extends the antiviral role of IFITMs to adaptive immune cells [109,110]. Two recent studies highlight broader roles of IFITMs in host immune pathways beyond antiviral activity. IFITM3 has been shown to be critical in phosphoinositide 3-kinase (PI3 K) signal amplification for the rapid expansion of B cells with high affinity to antigen [106]. IFITM3 has also been implicated in neuroinflammation as it modulates amyloid-β production by regulating γ-secretase activity [111].

Palmitoylome profiling of mouse dendritic cells identified IFITM3 *S*-palmitoylation [75]. IFITM3 is *S*-palmitoylated at three Cys residues (Cys71, C72 and 105) and loss of *S*-palmitoylation leads to abrogation of IFITM3 antiviral activity against influenza virus (figure 5*b*), and similar loss of antiviral activity was reported for palmitoylation-deficient IFITM1 [112]. Further APE analysis showed that Cys72 is the major site of modification of IFITM3 and mutation of this residue significantly lowers antiviral activity [35]. Also, mass spectrometric analysis of purified IFITM3 from mammalian cells revealed Cys72 as the predominant site of modification [113]. Cys72 is highly conserved across most mammals and is required for the antiviral activity of IFITM3 orthologues from mice, bats and humans [114,115]. Indeed, palmitoylation of evolutionarily ancient IFITM homologues present in mycobacteria were found to be palmitoylated when expressed in human cells [116]. Site-directed mutagenesis and live-cell imaging studies showed that Cys72 is essential for IFITM3 trafficking and colocalization with influenza virus in the endocytic pathway [117,118]. Recent NMR studies with IFITM3 chemically modified at Cys72 with maleimide-palmitate show that lipid modification at Cys72 stabilizes IFITM3 amphipathic helix membrane interaction which is important for restriction of virus infection [44,119]. These studies demonstrate the importance of site-specific palmitoylation of IFITMs in their antiviral activity. Overexpression of multiple palmitoyltransferases (DHHC 3, 7, 15 and 20) leads to increased IFITM3 S-palmitoylation but DHHC20 might be the most important for IFITM3 antiviral activity [120]. Roles for palmitoylation of IFITM3 in PI3 K signalling and regulation of γ-secretase activity remain to be investigated.

#### 3.2.3. TNF signalling

Tumour necrosis factors (TNFs) form another important superfamily of cytokines. TNF*α* regulates a variety of cellular processes including inflammation, proliferation, differentiation, and can induce various forms of cell death. TNF is synthesized as a transmembrane protein (tmTNF) and presented at the plasma membrane where it is cleaved and released as soluble TNF (sTNF). Membrane-bound N-terminal fragment of TNF (NTF) is further cleaved by signal peptide peptidase-like 2b to generate intracellular domain of TNF*α* (ICD-TNF*α*). All the TNF forms show biological activities. Metabolic labelling with [^3^H]palmitic acid identified *S*-palmitoylation of tmTNF [76]. Recent studies indicate a role of palmitoylation in tmTNF lipid raft partitioning, NTF stability and efficient cleavage for ICD-TNF*α* formation [121]. Interaction of *S*-palmitoylated tmTNF with TNFR1 in lipid rafts diminishes sensitivity to sTNF, thus regulating downstream NFkB and ERK1/2 signalling pathway.

Dynamic *S*-palmitoylation of TNFR1 also regulates TNF signalling [74]. Plasma membrane TNFR1 activation leads to recruitment of complex 1 adapter proteins to TNFR leading to NF-κB signalling. On the other hand, K63-ubiquitylation and internalization of TNFR1 triggers apoptosis signalling. TNFR1 can be palmitoylated at multiple Cys but dynamic *S*-palmitoylation at transmembrane proximal Cys248 regulates its plasma membrane localization. Depalmitoylation of activated TNFR1 by APT2 is necessary for enhanced NF-κB signalling, whereas knockdown of APT2 enhances Caspase-8 mediated cell death and reduced NF-κB signalling. Other members of TNF-receptor family have also been reported to be palmitoylated. *S*-palmitoylation of DR4 is promotes lipid raft association and cell death signalling, whereas DR6 *S*-palmitoylation prevents lipid raft association [77,78,122].

## 4. Conclusion and perspective

Over the past decade, we have developed greater understanding of *S*-palmitoylation-dependent regulation of proteins in innate and adaptive immune signalling. This is a testament to the development of chemical tools to study *S*-palmitoylation. Use of these chemical tools and proteomics methods allowed *S*-palmitoylome profiling of different cell types and helped in development of *in silico* prediction of *S*-palmitoylation sites in proteins. Further use of chemical inhibitors and genetic methods helped to understand the role of *S*-palmitoylation in the regulation of protein function. Coupling of these tools has led to the discovery of novel protein *S*-palmitoylation and mechanisms of protein regulation. However, it should be noted that functional analysis remains to be performed for many putative palmitoylated proteins identified in large scale *S*-palmitoylome profiling studies, making this a rich area for future investigations.

Though we have learned significantly about *S*-palmitoylation, we still have very little understanding of the implications of a dynamic lipid modification of proteins in cell signalling and regulation in comparison to other dynamic modifications like phosphorylation or ubiquitination.

We have little understanding of the mechanism and substrate specificity of the writers and erasers of palmitoylation. Structural characterization of individual DHHCs and DHHC selective inhibitor development will be necessary future advances.

In recent years, we have recognized that the role of *S*-palmitoylation and modifying enzymes in immune cells extends beyond antimicrobial functions to cancer and autoimmune diseases, among others. Moreover, there has been an increasing understanding of their potential as an attractive target for therapeutic interventions, as has been discussed in this review.

## Data accessibility

This article has no additional data.
